# Obstructive sleep apnea exaggeration as predictor of poor outcome post thrombolytic stroke

**DOI:** 10.1016/j.radcr.2024.04.069

**Published:** 2024-05-21

**Authors:** Rakhmad Hidayat, Ramadhanti Salma Ulwanda, Aruni Cahya Irfannadhira, Elvie Zulka Kautzia Rachmawati, David Pangeran, Reyhan Eddy Yunus, Taufik Mesiano, Mohammad Kurniawan, Al Rasyid, Salim Harris

**Affiliations:** a Faculty of Medicine Universitas Indonesia, Indonesia; b Dr. Cipto Mangunkusumo Hospital, Indonesia

**Keywords:** Stroke, Obstructive sleep apnea, CT perfusion, Thrombolysis, rtPA

## Abstract

Obstructive sleep apnea (OSA) is a common sleep disordered breathing in stroke patients. This case report aimed to show the presence of OSA in stroke can contribute to the increasing chance of mortality and morbidity. We presented a case of first-time stroke in a 64-year-old female with a history of pre-stroke OSA. She underwent intravenous thrombolysis as main therapy within the time limit under 4.5 hours since the stroke onset. She had prolonged hospital stay due to complications from OSA, even though she only had a small ischemic core (9 mL) in follow-up radiological imaging and was discharged with a greater National Institutes of Health Stroke Scale (NIHSS) score than admission (5 to 10). OSA can be one of warning signs for poor prognosis in stroke patients. Understanding the presence of OSA not only can be beneficial toward choosing the next steps of therapy, but also important for the rehabilitation and recovery period of stroke patients.

## Introduction

Stroke is one of the main causes of death and disability in the world with approximately 12.2 million new cases happen every year, and over 62% of them are ischemic cases with approximately 3.3 million deaths annually [1]. Obstructive sleep apnea (OSA) has been contributing as a risk factor in both cardio and cerebrovascular diseases, including stroke [2,3]. The studies shows that independently associated with neurocognitive and outcomes and type 2 diabetes [4,5].

It is a condition in which partial or complete airway obstructions happen during sleep, causing a case of hypoxia and sleep discontinuity [6,7]. Whether it appears prior to stroke or as a consequence of stroke, OSA has been associated with increase of stroke morbidity and mortality [8]. Here, we reported a case of a first time ischemic stroke with OSA as one of the comorbidities resulting in clinical deterioration during hospital stay.

## Case report

A 64-years-old woman came to the emergency room, Dr. Cipto Mangunkusumo General Hospital, Jakarta, presented with acute right-sided weakness (onset of 3.5 hours) followed by a case of dysarthria. She had class III obesity (BMI 42.1 kg/m^2^), a 10 years history of hypertension and 5 years of type 2 diabetes mellitus. All of them were treated with amlodipine 5 mg once daily and metformin 500 mg once daily.

On examination, the patient was compos-mentis, and had initial blood pressure 190/110 mmHg. Neurological examination showed seventh and 12th type cranial nerve palsy and slight hemiparesis on the right side (4 from maximum 5). The National Institutes of Health Stroke Scale (NIHSS) score on admission was 5. Routine laboratory tests were within the normal limits, except for hyperglycemia (blood sugar level 322 mg/dL).

**Fig. 1:**
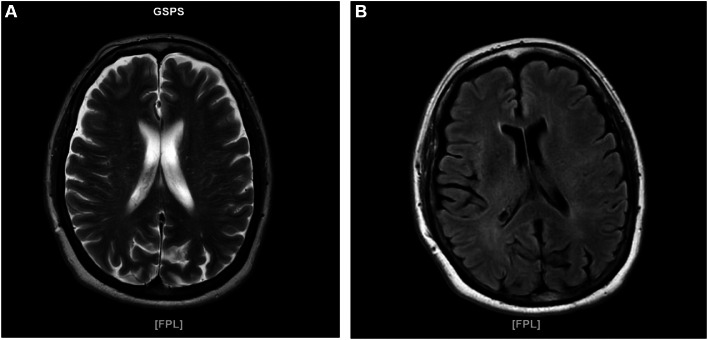
Non contrast MRI in T2W (A) and T2-FLAIR (B) showed no sign of bleeding to exclude the thrombolysis contraindication.

**Fig. 2:**
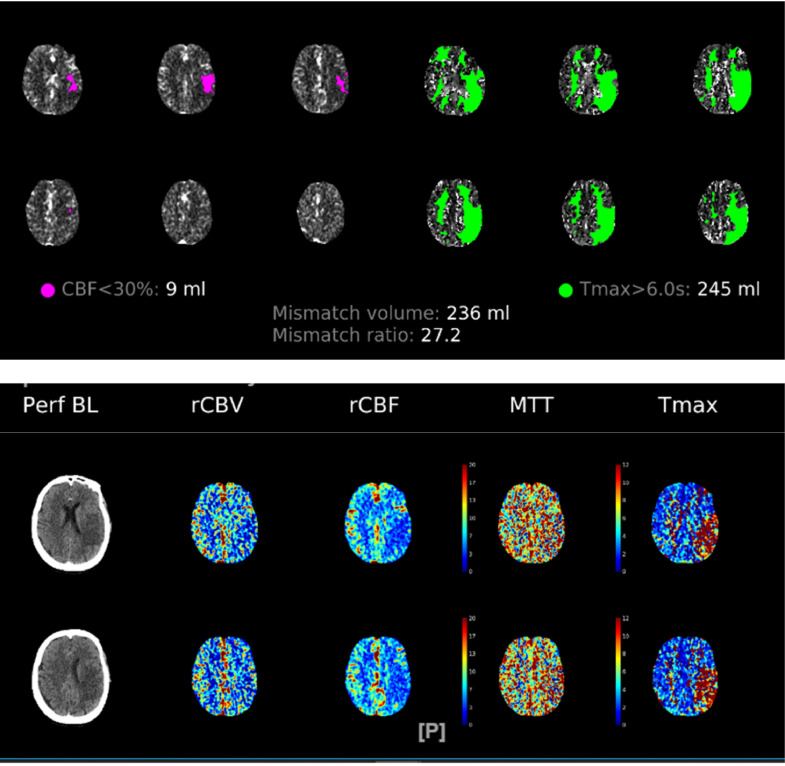
Automated brain computed tomography perfusion imaging, hypoperfusion area is seen in the infarcted region in the left frontoparietotemporal region (CBF<30% [9] in corresponding to 9 mL ischemic core, and Tmax > 6s accounts for hypoperfusion area of 245 mL, with mismatch volume of 236 mL and ratio of 27.2).

Initial radiological imaging using non-contrast Magnetic Resonance Imaging (MRI) showed no bleeding or space occupying lesion (Fig. 1). And an infarcted area in the left frontoparietotemporal region was shown in the follow-up computed tomography perfusion (CTP, Fig. 2). She was treated with nicardipine infusion to control her blood pressure and thrombolytic was conducted with dosage of (0,7)g/kg. NIHSS 1 hour after thrombolytic is getting better.

On the third day of hospitalization, she experienced a decrease of consciousness, but was quick to recover. Therefore, a repeat CT perfusion was performed and an occlusion of the left M2 was found. She was consulted with an otorhinolaryngologist and diagnosed with OSA. A polysomnography showed moderate OSA with apnea-hypopnea index (AHI) 27.5/ hr, with variation of oxygen saturation between 83 and 98% during the sleep test. She was moved to intensive care unit (ICU) and admitted for 17 days, using high-flow-nasal cannula (HFNC; Fi02 60%, flow 50 lpm) tapered down in tune with clinical condition. She was hospitalized for 42 days due to complications, and was discharged with NIHSS of 10.

## Discussion

The main therapy used in acute ischemic stroke (AIS) within the eligibility criteria is intravenous thrombolysis using alteplase [10]. We present a case of first time stroke admitted to the hospital. It fulfilled the eligibility of thrombolysis with no contraindication; thus the treatment was conducted. In our center, thrombolysis was conducted in patients with onset of stroke <6 hours with dosage of 0.6-0.9 mg/kg depending on the time of admission and the patient's weight, in our case it was given with dosage of 0.7 mg/kg.

Radiological imaging plays an important role in both diagnosis and the possibility of IV alteplase administration, with both noncontrast CT (NCCT) and magnetic resonance imaging (MRI) are considered effective choices to exclude intracerebral hemorrhage before thrombolysis [10,11]. The initial imaging used in our case was 6 minutes non contrast MRI-MRA protocol which included 5 steps of protocol: diffusion-weighted imaging (DWI), EPI-fluid attenuation inversion recovery imaging (FLAIR), EPI-gradient recalled echo (GRE), contrast-enhanced MRA (CE-MRA), dan dynamic susceptibility contrast (DSC) perfusion imaging [12]. We use another imaging that is beneficial in stroke that was used in this case is CTP. It helps to visualize parenchymal infarction from perfusion changes and whether it can be spared with reperfusion [11,^13^,14], thus, providing information about prognosis and further treatment decisions. CTP was used in this case to oversee the progression after thrombolysis on the second day. CTP itself is beneficial in the selection of candidates for mechanical thrombectomy between 6 and 24 hours after the onset of stroke, but in our case, CTP was used to comprehend the cerebral perfusion.

The case called for our interest because regardless of intravenous alteplase administration established within the time limit of ≤4.5 hours, and radiological imaging showed only a small irreversible ischemic core in CTP (9 mL), the patient still had poor clinical outcome.

There are several factors that can predict the outcomes of stroke patients. Age ≥65 years old and initial stroke severity are major risk factors that affect both mortality and morbidity within the first year in first time stroke, and both also play significant factors of neurological deterioration (ND) [12]. It can also be noted that high initial glucose level in the case can be related with ND due to the stress hyperglycemia mechanism [15,16].

Another factor that can be included was the presence of OSA in the patient. Sleep disordered breathing (SDB) is a common occurrence in stroke, accounting for about 50–80% in stroke patients, with OSA as the most common variety [17,18]. In our case, the patient had a pre-existing history of snoring while sleeping before the onset of stroke. The polysomnography conducted during hospitalization periods classified the patient with moderate-severe OSA with AHI 27.5/ hour. OSA has been associated with degree of severity in stroke; OSA with AHI ≥25 is connected with more neurological deficits and worsening the severity of stroke [8].

A study between OSA and non-OSA groups in stroke showed no significant difference in mean NIHSS scores at admission, but mean NIHSS scores at discharge are significantly higher in OSA than in non-OSA groups (8.15 vs 5.25, *P* = 0.002) [19]. This study was in line with our case, in which the patient had higher discharge NIHSS compared on admission. Another study denoted the likelihood of OSA happening in stroke patients treated with thrombolysis; with more severe degree of OSA in the thrombolysis group compared to non-thrombolysis one [20]. It might correlate due to the significant increase of inflammatory markers in OSA, causing systemic inflammatory response syndrome (SIRS) [5,20]. After intravenous thrombolysis, the existence of SIRS is shown to be associated with low short-term functional outcome [21].

Whether it appears before the onset of stroke or after, OSA can worsen disabilities by reducing cerebral perfusion in consequence of recurrent hypoxia [21]. The main modality to treat OSA is by using continuous positive airway pressure (CPAP). The use of HFNC is similar to CPAP in regards to both of them generating positive airway pressure. In our case, there was a case of dysphagia. Dysphagic stroke patients with nasogastric tubes can cause disturbance of nasal CPAP due to air leakage, therefore HFNC can be the alternative for treatment. HFNC is effective at reducing OSA severity in post-acute ischemic stroke patients especially in severe OSA. HFNC can improve oxygen desaturation index (ODI) and minimum Sp0_2_ level [22].

A case of hypoxia might explain the short period of loss of consciousness in the patient during the hospitalization. Pre-existing OSA might also increase the risk of adverse outcome in the patient, in both morbidity and mortality. With repeated hypoxia and apnea taken effects on the hypoperfusion area around the ischemic core and might result in more neuronal damage, and consequently, poor clinical outcome.

## Conclusion

OSA can be one of warning signs for poor prognosis in stroke patients. Understanding the presence of OSA not only can be beneficial toward choosing the next steps of therapy, but also important for the rehabilitation and recovery period of stroke patients. A clinician's awareness of pre-existing OSA diagnosis should not hinder the decision of intravenous alteplase administration, for reperfusion and revascularization with intravenous thrombolysis are time-dependent; an earlier approach can potentially reduce the poor clinical outcome.

## Ethics approval and consent to participate

Not applicable.

## Consent for publication

Verbal and written informed consents were given by the patient for the publication of this case.

## Authors’ contributions

All authors attest that they meet the current ICMJE criteria for authorship. RH and RS conceptualized and designed the study. RS and AC drafted the initial manuscript. RH, EZ, RS, DP, TM, and RE reviewed and revised the manuscript, final editing of the manuscript. RH, MK, AR, and SH critically reviewed the manuscript for important intellectual content. All authors approved the final manuscript as submitted and agree to be accountable for all aspects of the work. All authors have critically reviewed and approved the final draft and are responsible for the content and similarity index of the manuscript.

## Patient consent

In this case report, The patient signed a written informed consent that authorizes the research team to report the necessary information before data collection. Explanation of the case and reason for publication was thoroughly explained by the lead Physician (Consultant Neurologist) taking care of the patient.
